# Clinical non-penetrance associated with biallelic mutations in the RNase H2 complex

**DOI:** 10.1007/s10875-023-01438-2

**Published:** 2023-01-27

**Authors:** Yanick J Crow, for the AGS group, Luis I Gonzalez-Granado, Giulia Coarelli, Lucie Pierron, Isabelle Maystadt, Matias Wagner, Mina Zamani, Saeid Sadeghian, Clémence David, Gillian I Rice

**Affiliations:** 3 Unidad de Inmunodeficiencias, Pediatría, Instituto de Investigación Hospital 12 de Octubre, Facultad de Medicina, Universidad Complutense, Madrid, Spain; 4 Sorbonne Université, Paris Brain Institute - ICM, Inserm, CNRS, APHP, Paris, France; 5 Department of Genetics, Neurogene National Reference Centre for Rare Diseases, Pitié-Salpêtrière University Hospital, Assistance Publique, Hôpitaux de Paris, Paris, France; 6 Centre de Génétique Humaine, Institut de Pathologie et de Génétique, Gosselies, Belgium; 7 Faculté de Médecine, URPhyM, UNamur, Namur, Belgium; 8 Institute of Human Genetics, Klinikum rechts der Isar, School of Medicine, Technical University of Munich, Munich, Germany; 9 Institute for Neurogenomics, Helmholtz Zentrum München, Neuherberg, Germany; 10 Division of Pediatric Neurology, Developmental Neurology and Social Pediatrics, Dr. von Hauner Children’s Hospital, Munich, Germany; 11 Department of Biology, Faculty of Science, Shahid Chamran University of Ahvaz, Ahvaz, Iran; 12 Narges Medical Genetics and Prenatal Diagnosis Laboratory, East Mihan Ave, Kianpars, Ahvaz, Iran; 13 Department of Pediatric Neurology, Golestan Medical, Educational, and Research Center, Ahvaz Jundishapur University of Medical Sciences, Ahvaz, Iran; 14 Division of Evolution and Genomic Sciences, School of Biological Sciences, Faculty of Biology, Medicine and Health, University of Manchester, Manchester Academic Health Science Centre, Manchester, UK; 1 MRC Human Genetics Unit, Institute of Genetics and Cancer, University of Edinburgh, Edinburgh, UK; 2 Laboratory of Neurogenetics and Neuroinflammation, Institut Imagine, Université de Paris, Paris, France

To the Editor

The p.(Ala177Thr) (A177T; c.529G>A) missense substitution in RNASEH2B is the most frequently identified mutation in patients with the type I interferonopathy Aicardi-Goutières syndrome (AGS). This mutation can cause disease in the homozygous or compound heterozygous state, with 97 probands found to carry the variant (48 homozygotes; 49 compound heterozygotes) in a pan-ethnic cohort of 107 families with RNASEH2B-related disease [1]. There are 386 individuals heterozygous for the A177T substitution (and no homozygotes) recorded on gnomAD (out of a total of 282,704 alleles), making it the second-most frequently observed AGS-associated mutation seen in controls (after the p.(Pro193Ala) mutation in ADAR, present in gnomAD on 606 of 282,848 alleles with a single recorded homozygote). The A177T mutation has been shown to reduce the enzymatic activity of the RNase H2 complex by disrupting the interface between an RNASEH2B α-helix and the RNASEH2C kinked helix. Loss of RNase H2 enzymatic activity is thought to result in the aberrant accumulation of RNA:DNA hybrids, and/or by-products of increased DNA damage, which then induce type I interferon signalling though the cyclic GMP-AMP synthase (cGAS)-stimulator of interferon genes (STING) pathway.

Mutations in RNASEH2B most characteristically manifest in the first few months of life as a subacute encephalopathy. However, biallelic mutations in RNASEH2B, including homozygosity for the A177T mutation, have also been reported in patients with isolated spastic paraparesis [1]. Notably, Briggs and colleagues described a female homozygous for the A177T mutation who presented with skin lesions characteristic of AGS for the first time at the age of 19 years, and who exhibited no features of neurological disease when she was last reviewed at 32 years of age [2].

Variable clinical expression is well recognised in AGS [1]. Furthermore, clinical non-penetrance has been described in relation to certain AGS genotypes. In particular, a study of MDA5-associated disease identified 10 of 74 individuals, 13.5% of all mutation carriers ascertained, to be clinically asymptomatic (with seven of these aged over 50 years) [3]. However, while clinical non-penetrance is well-appreciated in the context of dominantly inherited AGS (MDA5 discussed immediately above, and see also the recurrent p.G1007R dominant-negative mutation in ADAR1, and certain other type I interferonopathies (most particularly COPA syndrome)), the frequency of clinical non-penetrance related to AGS genotypes inherited as an autosomal recessive trait is unknown, because the clinically asymptomatic siblings of an affected child are not normally characterised molecularly.

Here, we report 4 clinically asymptomatic individuals homozygous for the A177T mutation in RNASEH2B, and a further asymptomatic individual with biallelic mutations in RNASEH2A.

We identified 5 families in which one individual was found to be clinically asymptomatic into adulthood (age range 18 – 68 years) (Table 1). Three parents were ascertained in the course of family testing following the diagnosis of classical AGS (2) or spastic paraparesis (1) in their affected offspring. One individual was the older sibling of a child with classical AGS. Finally, a female was identified incidentally as homozygous for the A177T mutation following trio exome sequencing instigated because of disease in her child unrelated to AGS. In the two individuals where cerebral computed tomography (CT) was performed, the result was normal, with no evidence of calcification. Interferon signalling, assessed in the blood of two asymptomatic parents, was normal in one case (at age 68 years), and minimally elevated in the other (at 37 years of age). Of note, 20-30% of patients with biallelic mutations in RNASEH2B do not demonstrate a significant upregulation of interferon signalling in blood, and a lower median ‘interferon score’ is observed in patients with mutations in RNASEH2B compared to other AGS-associated genotypes [1].

In light of ongoing efforts to develop treatments for AGS [4], the observation of individuals clinically asymptomatic into adulthood is important when considering the assessment of therapeutic efficacy in future clinical trials. This point is of particular note given that mutations in RNASEH2B represent the most frequent genetic cause of AGS. Additionally, this finding is relevant to the possibility of neonatal screening for AGS [5]. The question remains as to whether such individuals are at risk of developing disease over time, and/or at what age one might be reassuring that disease will not develop. The observation of a 68-year-old clinically asymptomatic individual, with no evidence of intracranial calcification and normal expression of interferon stimulated genes (ISGs) in blood, indicates that homozygosity for the A177T mutation is compatible with disease-free status over the long-term. Why such individuals remain asymptomatic is unclear, but presumably relates to other genetic (‘protective’ or ‘susceptibility’ alleles in these individuals or their affected relatives respectively) or epigenetic factors, and/or an absence of exposure to relevant environmental triggers. More generally, it is clear that the absence of clinical disease does not rule out the possibility of an individual harbouring biallelic pathogenic mutations in RNASEH2A or RNASEH2B.

## Figures and Tables

**Table 1:** Summary clinical and molecular data

Family	AGS2776	AGS3366	AGS3434	MW1	MZ1
**Gene**	RNASEH2B	RNASEH2B	RNASEH2B	RNASEH2B	RNASEH2A
**Mutation(s)**	A177T Hom	A177T Hom	A177T Hom	A177T Hom	R186G Hom**
Proband*	M. Classical AGS (now aged 5)	F. Early onset, slowly progressive SP (now aged 32)	F. Normal development until age 1, and then classical AGS-like regression (now aged 3)	Child with developmental delay unrelated to AGS	M. Early onset SP (now aged 3)
**Asymptomatic family member**	Brother (age 19)	Mother (age 68)	Mother (age 37)	Mother (age 36)	Father (age 28)
**Other**	Normal cerebral CT in asymptomatic brother (at age 2)	Normal cerebral CT in mother aged 68. IS normal in the proband and mother (ages 32 and 68 respectively)	IS: 3.09 (mother) and 4.61 (child) (at ages 37.32 and 2.84)	None	None

* All ages given in years

** c.557G>A / p.(Arg186Gln); CADD = 29.5; Predicted damaging by PolyPhen2 and SIFT; Seen on 1/251,488 alleles on gnomAD; Also observed in AGS680 (south East Asian family) in combination with c.306del p.Thr103Profs*11, and in the homozygous state in an unreported child of Iranian ancestry experiencing regression at age 1.5 years and now demonstrating spasticity and no speech at age 3 years (i.e. a phenotype consistent with Aicardi-Goutières syndrome)

AGS = Aicardi-Goutières syndrome; CT = Computed tomography; F = Female; Hom = Homozygous; IS = Interferon score derived from the expression of 24 ISGs in blood measured on a NanoString platform (normal < 2.76); M = Male; SP = Spastic paraparesis
